# Purification and Oxidative Scavenging of Total Alkaloids of *Piperis longi fructus* Based on Adsorption Kinetics and Thermodynamic Theory

**DOI:** 10.3390/molecules30071476

**Published:** 2025-03-26

**Authors:** Lirong Lu, Dezhi Shi, Nuo Chen, Chengchao Wu, Hang Zhang, Shaohui Zhong, Jing Ji, Yunfeng Zheng, Jianming Cheng, Shiwen Huang, Taoshi Liu

**Affiliations:** 1 College of Pharmacy, Nanjing University of Chinese Medicine, Nanjing 210023, China; 20220970@njucm.edu.cn (L.L.); 202430173@njucm.edu.cn (D.S.); 20220779@njucm.edu.cn (N.C.); 20220949@njucm.edu.cn (C.W.); 20230914@njucm.edu.cn (H.Z.); 20220799@njucm.edu.cn (S.Z.); jier4522@163.com (J.J.); zyunfeng@njucm.edu.cn (Y.Z.); 320320@njucm.edu.cn (J.C.); 2 Jiangsu Province Engineering Research Center of Classical Prescription, Nanjing 210023, China

**Keywords:** *Piperis longi fructus*, enrichment, adsorption kinetics, adsorption thermodynamics, antioxidant activity

## Abstract

An effective method for purifying the total alkaloid components from *Piperis longi fructus* extract was developed in this study. The adsorption/desorption processes of the total alkaloid components from *Piperis longi fructus* were established by resin model screening, adsorption kinetics, and adsorption thermodynamics tests. Moreover, the purified powders were analyzed with UPLC-Q-ZENO-TOF-MS/MS and then their antioxidant activity was tested. The Langmuir equation provided a good fit with the experimental results. The thermodynamic study provides a satisfactory fit for the isotherm data, indicating that the adsorption process is characterized by spontaneity (Δ*G*° < 0), exothermicity (Δ*H*° < 0), and an increase in entropy (Δ*S*° < 0). Furthermore, the kinetic adsorption behavior on D101 resin was effectively modeled by pseudo-second-order kinetics. According to this mechanism, we selected the best adsorption parameters and optimized the on-column elution process to effectively enrich the total alkaloid components. The optimal process was as follows: D101 macroporous resin was added to an alcohol solution (crude drug concentration of 2 g/mL) and then concentrated under a vacuum at 45~55 °C (<−0.08~−0.10 MPa) until alcohol-free. Subsequently, the resin was loaded into the column and eluted with 70% ethanol at a flow rate of 2 BV/h for 10 BV to achieve desorption. The present study provides a more efficient method for the enrichment of the total alkaloidal components of *Piperis longi fructus*, which will lay the foundation for applications in food additives or functional foods in the future.

## 1. Introduction

*Piperis longi fructus*, a traditional Chinese medicinal and edible plant, belongs to the Piper genus of the Piperaceae family [1]. This species is indigenous to the Indo-Malaya region and is extensively distributed across tropical and subtropical areas, encompassing the Indian subcontinent, Sri Lanka, the Middle East, and the Americas [2]. The fruits of *Piper longum* L., known as long pepper, are highly valued for their culinary uses and are employed as a seasoning in cooking and food processing [3]. As a flavoring agent, they can be used together with other spices to pickle meat or as a base material [4]. *Piperis longi fructus* is rich in amide alkaloids, which exhibit anti-inflammatory [5], antioxidative, cardiovascular protection, and other pharmacological effects [6]. High contents of piperine have a strong antioxidant effect. Research indicates that piperine may play a role as an antioxidant by influencing the PMNL 5-lipoxygenase (5-LO) pathway [7]. Additionally, *Piperis longi fructus* has demonstrated neuroprotective effects, as evidenced by the anxiolytic and antidepressant-like effects of its methanolic extract in a beta-amyloid rat model of Alzheimer’s disease [8].

Macroporous resin is a kind of organic polymer which is insoluble in acid, alkali, and various organic solvents [9]. Its mechanism combines adsorption and molecular sieving to selectively adsorb targeted substances and remove impurities [10]. In recent years, the application of macroporous resin technology has become increasingly prevalent in the purification processes of various components and active ingredients found in traditional Chinese medicine. It has the characteristics of good selectivity, high mechanical strength, convenient regeneration, and fast adsorption speed [11]. At present, some scholars have extracted piperine from *Piperis longi fructus* using supercritical extraction technology [12]. However, there is no research on the adsorption mechanism of total alkaloids on macroporous resin. In this study, the purification effects of several common macroporous adsorption resins on the total alkaloids of *Piperis longi fructus* were compared. The mechanism of adsorption of alkaloids by the macroporous adsorption resin was analyzed by adsorption kinetics and thermodynamics, and the purification process on the column was optimized. This provides a theoretical basis for the industrial production of the total alkaloids of piper longum by resin adsorption. In addition, the purified components were characterized by UPLC-Q-ZENO-TOF-MS/MS, and the antioxidant activity of the total alkaloids was compared before and after purification. This research provided a reference for the subsequent study of the alkaloid activity of *Piperis longi fructus*.

## 2. Results

### 2.1. Selection of the Best Macroporous Resin of Piperis longi fructus

**Figure 1:**
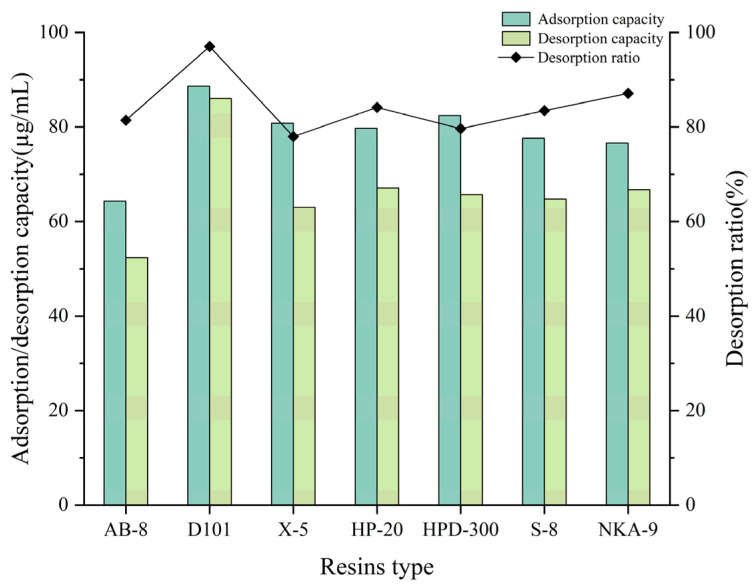
Results of adsorption/desorption capacity and desorption ratio of total alkaloids of seven resins.

The static adsorption/desorption properties of different resins were investigated to identify a suitable resin for the total alkaloids, with the results shown in Figure 1. It was observed that D101 macroporous resin exhibited favorable adsorption and desorption effects on the alkaloids. Therefore, the adsorption kinetics and thermodynamics of D101 macroporous resin for the alkaloids of *Piperis longi fructus* will be further studied.

### 2.2. Adsorption Kinetics of D101 Macroporous Resin of Piperis longi fructus

**Figure 2:**
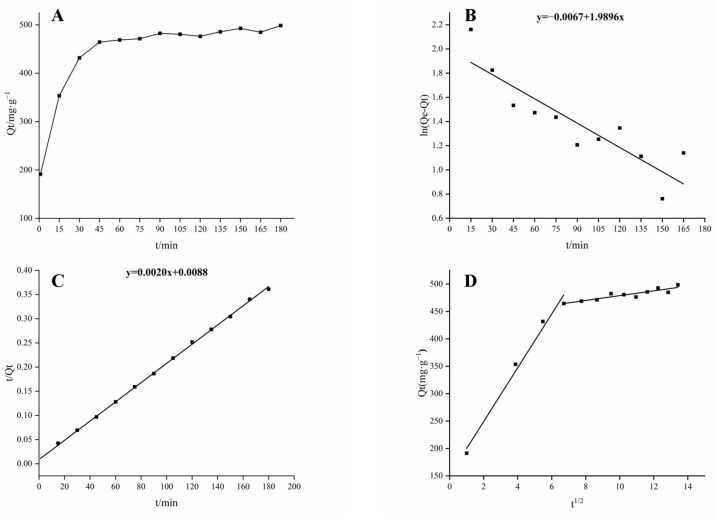
The results of adsorption kinetics. (**A**) Adsorption kinetic curves; (**B**) the pseudo-first-order model; (**C**) the pseudo-second-order model; (**D**) the Weber–Morris model. *Q_t_* represents the adsorption per unit of resin at the moment *t*; *Q_e_* represents the equilibrium adsorption per unit of resin.

In this study, three classic mathematical models were employed to investigate the adsorption principle of D101 macroporous resin for the total alkaloids. As depicted in Figure 2, the adsorption capacity exhibited an upward trend over time, while the rate of adsorption progressively decreased.

The kinetic analysis revealed that the *R*^2^ value for the pseudo-second-order kinetic model was above 0.9, indicating a high degree of model–data correlation. This finding implies that the pseudo-second-order kinetic model is particularly suitable for describing the adsorption dynamics of the total alkaloids onto D101 macroporous resin. The pseudo-second-order kinetic model implied that the adsorption rate of the resin was primarily influenced by chemical adsorption within the resin, mainly the electron sharing or exchange of electrons between the adsorbent and the substrate. The greater the consistency between the data and the model, the more significant the contribution of chemical adsorption to the overall adsorption process [13]. The intraparticle diffusion model includes the outer surface diffusion, the intraparticle diffusion, and the interaction between the substrate and the adsorption site [14]. The results indicated that *K*_d1_ > *K*_d2_, suggesting that the surface diffusion process of the resin is greater than the internal diffusion process [15]. The results of the three adsorption kinetic equation parameters are shown in Table 1.

### 2.3. Adsorption Thermodynamics of D101 Macroporous Resin with Piperis longi fructus

**Figure 3:**
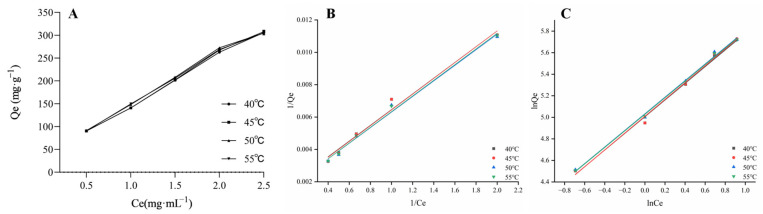
The results of adsorption thermodynamics. (**A**) Adsorption isotherms. (**B**) Langmuir models. (**C**) Freundlich models. Three models for total alkaloids on D101 resin at 40, 45, 50 and 55 °C. *Q_e_* represents the adsorption capacity at equilibrium; *C_e_* represents the equilibrium concentration.

The isotherm reflects the relationship between the adsorption equilibrium and the equilibrium adsorption amount [16]. As shown in Figure 3, the equilibrium adsorption amount on D101 macroporous resin increased with an increase in the concentration and temperature, indicating that the increase in temperature was beneficial to the adsorption of the total alkaloids of *Piperis longi fructus* on D101 macroporous resin, and the adsorption process was an endothermic process. The adsorption process was further described by the Freundlich equation and the Langmuir equation. The Langmuir isotherm model describes the monolayer adsorption on the resin without considering the interaction between adjacent adsorption molecules, while the Freundlich isotherm model is suitable for describing multilayer adsorption [17]. The fitting parameters of the two equations are shown in Table 2. In the Freundlich isotherm model, the parameter n is associated with the intensity of sorption, which reflects both the driving force behind the sorption process and the distribution of energy among the sorption sites [18]. It is difficult for adsorption to occur when the value of 1/n is between 0.5 and 1 [19]. It can be seen that the total alkaloids of *Piperis longi fructus* are more suitable for the Langmuir model, indicating that D101 macroporous resin is conducive to the adsorption process of total alkaloids of *Piperis longi fructus*.

The thermodynamic parameters are shown in Table 3; Δ$H^{o}>$0, Δ$G^{o}<0$, indicating that the adsorption is endothermic and spontaneous [20]. Δ$S^{o}>$0, indicating that the adsorption is an entropy-increasing process. In addition, the absolute value of Δ$G^{o}$ increases with the increase in temperature, indicating that higher temperatures are beneficial for adsorption, which is consistent with the change in the adsorption kinetic curve [21].

### 2.4. Results of On-Column Process Optimization

**Figure 4:**
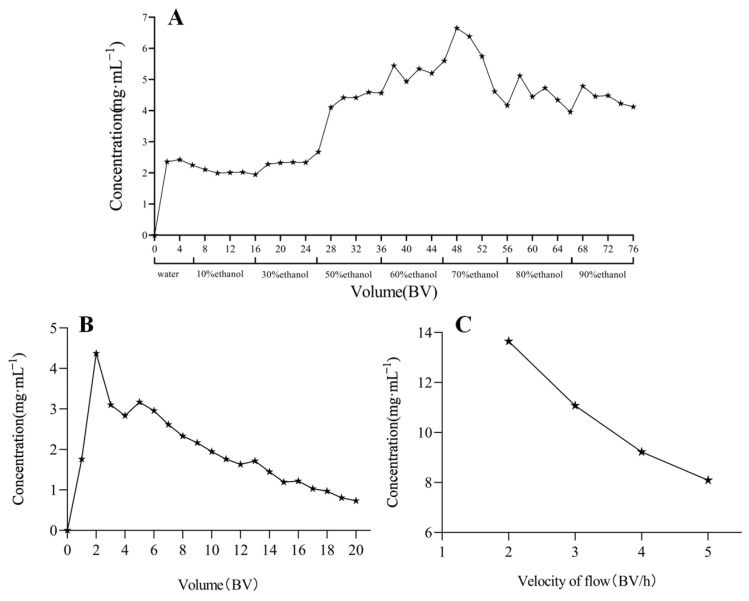
The results of the on-column process optimization. (**A**) Ethanol elution concentration study; (**B**) ethanol elution volume study; (**C**) elution flow rate study.

The specific results are shown in Figure 4. The findings regarding ethanol concentration indicated that as the ethanol concentration rose, the concentration of the sample also increased steadily. When the ethanol concentration reached 80%, the concentration began to decrease, indicating that the adsorption capacity of D101 macroporous resin to the total alkaloids reached saturation. Thus, 70% ethanol was selected as the elution concentration for the component.

The findings from the ethanol elution volume indicated that as the volume of ethanol increased, the concentration of the sample progressively diminished, indicating that the adsorbed components were gradually eluted. Although there were still some residues in the resin, the efficiency of continuous elution was reduced and tended to be smooth. Therefore, 10 BV of 70% ethanol was selected as the elution volume for the component.

The results of the flow rate investigation showed that a flow rate of 2 BV/h was selected as the elution flow rate for the total alkaloid component. Based on the above considerations, the column elution process was established as follows: 10 BV of 70% ethanol eluted at a flow rate of 2 BV/h.

The freeze-dried powder is shown in Supplementary Materials Figure S1. The yield of three batches of process validation was 1.73%, 1.71%, and 1.66%, respectively, with RSD values of 2.12%. The content of alkaloid after purification through D101 macroporous resin increased from 11.35% to 46.95%, and the recovery was 1.70%. The results of the three batches of validation showed that the preparation method was stable and reliable, while the determination of the content of total alkaloids was feasible.

### 2.5. Results of Component Analysis by UPLC-Q-ZENO-TOF-MS/MS

**Figure 5:**
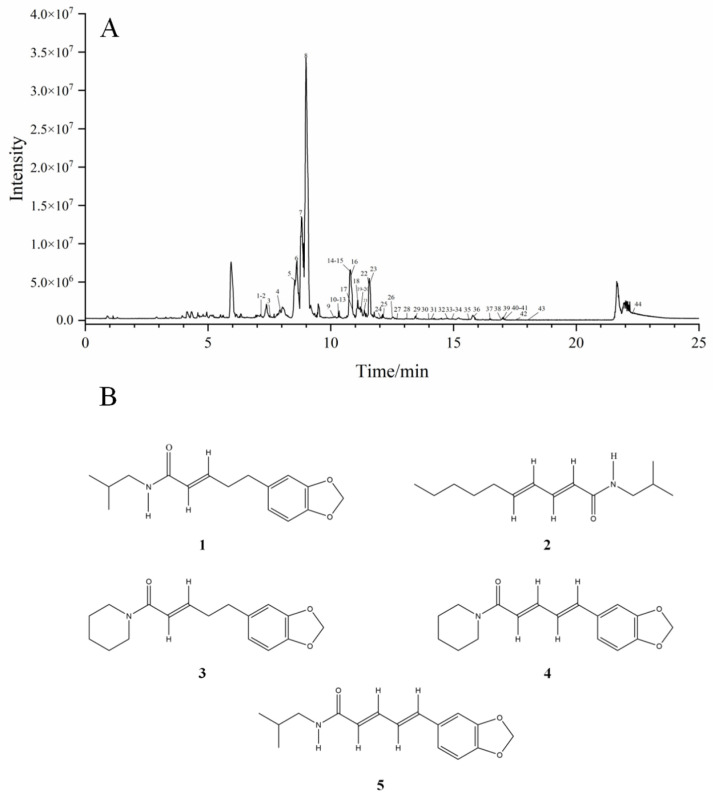
Results of the component analysis by UPLC-Q-ZENO-TOF-MS/MS. (**A**) The base peak chromatogram (BPC) of *Piperis longi fructus* in the positive-ion mode; (**B**) the structure of five high-content alkaloid components, where (**1**) is 4,5-dihydropiperlonguminine, (**2**) is pellitorine, (**3**) is piperanine, (**4**) is piperine, and (**5**) is piperlonguminine.

The total ion current diagram of *Piperis longi fructus* in positive ion mode and the structure of the total alkaloids are shown in Figure 5. Through UPLC-Q-ZENO-TOF-MS/MS component analysis, a total of 42 alkaloid compounds were identified, and the components are presented in Supplementary Materials Table S1. There were five high-content alkaloid components, mainly of the following three types. Type I compounds are synthesized via the condensation reaction between piperidine and unsaturated fatty acids that possess a benzodioxole tail group, such as piperanine, piperine, and piperettine I. Type II compounds are synthesized via the condensation reaction between isobutylamine and unsaturated fatty acids that possess a benzodioxole tail group, such as 4,5-dihydropiperlonguminine and piperlonguminine. Type III compounds are synthesized via the condensation reaction between isobutylamine and unsaturated fatty acids characterized by an n-pentyl tail group, such as pellitorine [22,23,24].

### 2.6. Results of Antioxidant Activity

**Figure 6:**
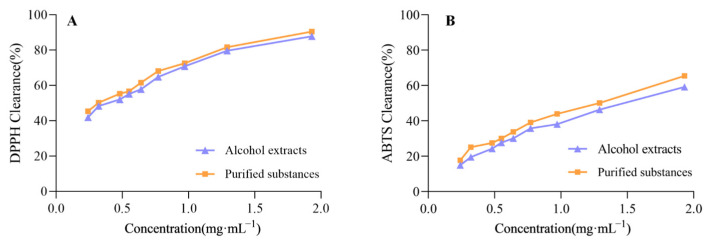
The results of the antioxidant activity of *Piperis longi fructus*. (**A**) Scavenging activity towards DPPH radicals; (**B**) scavenging activity towards ABTS^+^ radicals.

The scavenging activities of *Piperis longi fructus* on DPPH and ABTS^+^ radicals are illustrated in Figure 6. As the concentration of the sample increased, the scavenging rates of DPPH and ABTS^+^ exhibited a dose-dependent relationship. It can be observed from the results that the antioxidant activity after purification is better than that of the crude extract. The IC_50_ of DPPH and ABTS^+^ free radical scavenging was calculated by fitting the equation with the mass concentration of (X) and the inhibition rate of the crude extract and purified product as (Y). The linear equations are shown in Supplementary Materials Table S2. The DPPH and ABTS^+^ free radical scavenging were increased by about 1.5 times and 1.15 times after enrichment with D101 macroporous resin.

## 3. Discussion

In this study, we developed an effective method to purify the total alkaloids from *Piperis longi fructus* extracts. The adsorption mechanism of the total alkaloids from *Piperis longi fructus* on macroporous resin was studied through kinetic and thermodynamic equations. It was found through identification that the main components of the purified product are amide-type alkaloids. We intended to conduct a quantitative analysis of the components to compare whether this purification method could significantly increase the content compared to traditional separation methods. However, due to the tailing peaks and low sample loading often encountered in the separation of alkaloids, this research could not be carried out. Therefore, we supplemented the study with an evaluation of the antioxidant activity of the product before and after purification. The limitations of this study are that the analysis of the total alkaloid components is not comprehensive enough, and the influence of the selected temperature on the adsorption is not evident in the thermodynamic study. The follow-up plans are to increase the investigation of other temperatures and study the mechanism of anti-inflammatory and antioxidation of amide alkaloids in *Piperis longi fructus*.

## 4. Materials and Methods

### 4.1. Chemicals and Reagents

Analytical-grade ethanol was purchased from Sinopharm Chemical Reagent Co., Ltd. (Shanghai, China). Chromatography-grade acetonitrile and methanol were purchased from Zhejiang Yuexu Material Technology Co., Ltd. (Shanghai, China). *Piperis longi fructus* was purchased from Anhui Herb Market (Bozhou, China), and the samples were identified as the fruit of the *Piper longum* L. plant by Prof. Yan Hui (China), Faculty of Pharmacy, Nanjing University of Chinese Medicine (Nanjing, China). D101, HP-20, S-8, and AB-8 were purchased from Shanghai yuanye Bio-Technology Co., Ltd. (Shanghai, China), NKA-9, X-5, and HPD-300 were purchased from Beijing Solarbio Science & Technology Co., Ltd. (Beijing, China). Detailed information about resins is shown in Supplementary Materials Table S3. 

The SCIEX ZenoTOF 7600 High-Resolution Liquid Mass Spectrometer was purchased from AB SCIEX (Framingham, MA, USA). A multifunctional microplate reader was purchased from Nanjing Yanwo Bio-Technology Co., Ltd. (Nanjing, China).

### 4.2. Extraction of Crude Piperis longi fructus

A total of 200 g of *Piperis longi fructus* was taken and extracted twice by heating 20 times with 50% ethanol for 1 h and filtered, and then the lost weight was made up with 50% ethanol and shaken well. A total of 800 mL alcoholic extract was taken, and concentrations of 0.50, 1.00, 1.50, 2.00, and 2.50 g/mL were made up with 50% ethanol.

### 4.3. Quantification of Total Alkaloid Content

An appropriate volume of distilled water was measured and combined with concentrated sulfuric acid to prepare a 200 mL solution of 90% concentrated sulfuric acid. Subsequently, 0.20 g of chromotropic acid powder was added to the solution and dissolved, and the resultant mixture was stored in a brown bottle for airtight storage.

Test solution preparation: 200 μL of *Piperis longi fructus* ethanol extract was combined with 5 mL of chromic acid concentrated sulfuric acid solution, and then the mixture was mixed evenly. The mixture was heated at 100 °C for 30 min, cooled to room temperature, and diluted with pure water to 10 mL. The test solution absorbance was measured at 570 nm. The piperine reference substance was precisely measured and dissolved in methanol to prepare a stock solution of 0.26 mg/mL, which was further diluted to various concentrations. The content was determined by the establishment of a standard curve (Y = 0.0247 X + 0.0715, *R*^2^ = 0.9910), where Y signifies the absorbance and X represents the concentration of the reference substance.

### 4.4. Resin Screening

Approximately 1 g of each macroporous resin (AB-8, D101, X-5, HP-20, HPD-300, S-8, NKA-9) was placed into a 50 mL stoppered conical flask. Then, 20 mL of the sample solution of *Piperis longi fructus* was added and concentrated under a vacuum at 40 °C (<−0.08~−0.10 MPa) until alcohol-free. The solution was filtered and the filtrate was collected. The adsorption/desorption amount and desorption ratio were calculated by equations as follows (1)–(3):

(1) $$\mathrm{Adsorption}\;\mathrm{performance}:\;q_{1}=\frac{\left(C_{0}-C_{1}\right)V_{1}}{W}$$

(2) $$\mathrm{Desorption}\;\mathrm{performance}:\;q_{2}=\frac{C_{2}V_{2}}{W}$$

(3) $$\mathrm{Desorption}\;\mathrm{rate}:\;R=\frac{C_{2}V_{2}}{{\mathrm{Wq}}_{1}}\times 100\%$$

where *C*_0_ is the initial concentration of the solutes (mg/g), *q*_1_ and *q*_2_ are the adsorption capacities at adsorption equilibrium (mg/g resin), and *W* is the dry macroporous resin weight (g), which is equivalent to the mass *m*. *m* (g) is the dried mass of adsorbent used. C_1_ and *C*_2_ are the equilibrium concentrations of the adsorption and desorption solutions (mg/mL), and *V*_1_ and *V*_2_ are the volumes of the initial and desorption solutions (mL).

### 4.5. Adsorption Kinetics

The initial concentration of *the Piperis longi fructus* sample solution was set as 1 mg/mL for the kinetic adsorption studies. A total of 10 g of dry D101 macroporous resin was placed in a conical flask, to which 600 mL of the sample solution of *Piperis longi fructus* was added. Adsorption experiments were performed under a vacuum at 40 °C (<−0.08~−0.10 MPa) at 1, 15, 30, 45, 60, 75, 90, 105, 120, 135, 150, 165, and 180 min. The samples were taken at different times and analyzed separately. The adsorption kinetic curve was plotted with time (*t*) as the abscissa and adsorption capacity (*Q_t_*) as the ordinate [25]. The kinetic correlation equations are as follows (4)–(6):

(4) $$\mathrm{Pseudo}\text{-}\mathrm{first}\text{-}\mathrm{order}\;\mathrm{kinetic}\;\mathrm{model}\;\mathrm{equation}:\;\log\left(Q_{e}-Q_{t}\right)={\mathrm{logQ}}_{e}-\frac{K_{1}t}{2.303}$$

(5) $$\mathrm{Pseudo}\text{-}\mathrm{second}\text{-}\mathrm{order}\;\mathrm{kinetic}\;\mathrm{model}\;\mathrm{equation}:\;\frac{t}{Q_{t}}=\frac{1}{{K_{2}Q_{e}}^{2}}+\frac{t}{Q_{e}}$$

(6) $$\mathrm{Particle}\;\mathrm{diffusion}\;\mathrm{model}\;\mathrm{equation}:\;Q_{t}=K_{3}t^{1/2}+C$$

where *K*_1_, *K*_2_ and *K*_3_ are the rate constants for the pseudo-first-order kinetic model, the pseudo-second-order kinetic model and the intraparticle diffusion model. *Q_t_* corresponds to the adsorption per unit of resin at the moment *t* (mg/g resin), and *Q_e_* is the equilibrium adsorption per unit of resin (mg/g resin). *C* denotes the constant in the intraparticle diffusion models.

### 4.6. Adsorption Thermodynamics

A total of 20 mL of the sample solution of *Piperis longi fructus* with concentrations of 0.5, 1.0, 1.5, 2.0, and 2.5 g/mL of the raw drug was added to 1 g of the dry D101 resin. The samples of the five concentrations were processed under a vacuum at temperatures of 40, 45, 50, and 55 °C (<−0.08~−0.10 MPa) until alcohol-free, and then the adsorbed samples were taken up and assayed [26]. The thermodynamic equations are as follows (7)–(11):

(7) $$\mathrm{Langmuir}\;\mathrm{equation}:\;Q_{e}=\frac{Q_{m}K_{L}C_{e}}{1+K_{L}C_{e}}$$

where *Q_e_* is the adsorption capacity at equilibrium (mg/g), *K_L_* is the Langmuir constant, *C_e_* is the equilibrium concentration (mg/mL), and *Q_m_* is the maximum adsorption capacity of the resin (mg/g resin).

(8) $$\mathrm{Freundlich}\;\mathrm{equation}:\;{\mathrm{lnQ}}_{e}{\mathrm{lnK}}_{F}+\frac{1}{n}\ln C_{e}$$

where *K_F_* is the Freundlich constant that reflects the adsorption capacity, and 1/n is an empirical constant that indicates the magnitude of the adsorption driving force.

(9) $$\mathrm{Enthalpy}\;\mathrm{change}:\;{\mathrm{lnK}}_{L}=\frac{{∆S}^{o}}{R}-\frac{{∆H}^{o}}{\mathrm{RT}}$$

(10) $$\mathrm{Gibbs}\;\mathrm{free}\;\mathrm{energy}:\;{∆G}^{o}=-\mathrm{nRT}$$

where *R* is the universal gas constant and *T* is the absolute temperature.

(11) $${∆S}^{o}=({∆H}^{o}-{∆G}^{o})/T$$

where the values of ${∆H}^{o}$ are calculated from the slope and intercept of *lnC_e_* versus the 1*/T* curve.

### 4.7. On-Column Process Optimization

A total of 200 mL of the sample solution of *Piperis longi fructus* was added to 10 g of D101 dry resin and concentrated under a vacuum at 50 °C (<−0.08~−0.10 MPa) until alcohol-free. Subsequently, the mixture was loaded into a column and eluted at a flow rate of 2 BV/h. After the loading step, the column loaded with the adsorbed components was washed with deionized water at a flow rate of 2 BV/h to remove the unbound substances. This was followed by desorption using ethanol at varying concentrations to elute the targeted compounds [27]. The optimum elution solvent was determined through the above steps by examining the elution volume and elution rate (the column volume is approximately equal to 1.15 times the weight value of the dry resin).

### 4.8. Identification of Chemical Components by UPLC-Q-ZENO-TOF-MS/MS

Test solution preparation: The effluent was concentrated under a vacuum at 50 °C (<−0.08~−0.10 MPa) until alcohol-free, and the resulting aqueous solution was freeze-dried. Approximately 20 mg of the freeze-dried powder was accurately measured, and the sample was then placed into a conical flask equipped with a stopper. Subsequently, 50 mL of 50% ethanol was added to the conical flask, which was sealed and weighed. Then, it was treated with ultrasound (40 KHZ, 100 W) for 30 min. At the end of the ultrasound, the conical flask was removed and cooled to room temperature. The lost weight was made up with 50% ethanol, shaken well, and then filtered. The filtrate was taken and passed through a 0.22 μm microporous filter head.

Chromatographic conditions and mass spectrometric conditions: The detection was performed on a high-resolution liquid mass spectrometer ZenoTOF 7600(AB SCIEX, Framingham, MA, USA), and a column of Agilent polaris 3 C18A (100 mm × 2.0 mm, 3.0 μm, Agilent Technologies, CA, USA) was selected for the analysis [28]. The column was subjected to elution utilizing water as mobile phase A and acetonitrile as mobile phase B, following the specified gradient: 0~15 min, 10–90% B; 15~20 min, 90% B; 20~22 min, 90~10% B; 22~25 min, 10% B. Flow rate 0.3 mL/min; injection volume 3 μL; column temperature 40 °C. The MS conditions in the positive ion mode were set as follows: ion source temperature, 550 °C; flow rate of curtain gas, 35 psi; flow rate of nebulization gas and flow rate of auxiliary gas, 55 psi; ion spray floating voltage, 5500 V; collision energy, 35 eV; declustering potential, 100 eV. Data were acquired from 80 to 1250 Da for each sample. The sample acquisition time was 25 min.

### 4.9. DPPH and ABTS^+^ Antioxidant Activity

DPPH free radical-scavenging assay: A total of 40 mg DPPH was weighed accurately, dissolved in 50% ethanol, fixed in a 100 mL volumetric bottle (concentration of 0.04% DPPH), and preserved in darkness [29]. Test solutions with concentrations of 0.24 to 1.93 mg/mL (1 mL) were prepared and mixed with freshly prepared DPPH solution (1 mL). The mixture was reacted in a water bath at 37 °C for 20 min in darkness, after which the absorbance was measured at 517 nm.

ABTS^+^ assay: ABTS^+^ radicals were generated by dissolving 8.16 mg of ABTS and 1.4 mg of potassium persulfate (K_2_S_2_O_8_) in 2 mL of distilled water. Subsequently, 1 mL of each solution was thoroughly mixed and stored in darkness for 12 h. Before use, the solution was diluted with 50% ethanol until its absorbance at 734 nm was 0.700 ± 0.005. The test solutions (1 mL, 0.24 to 1.93 mg/mL) were combined with the freshly prepared ABTS solution (4 mL) and reacted in a water bath at 37 °C for 6 min in darkness, and the absorbance was measured at 734 nm [30]. The scavenging rate was calculated according to Formula (12), and the antioxidant activity of the sample was quantified by the concentration (IC_50_) with a 50% scavenging effect.

(12) $$\mathrm{Scavenging}\;\mathrm{rate}\;(\%)=(1-\frac{A_{1}-A_{2}}{A_{0}})\times 100\%$$

where *A*_1_, *A*_2_ and *A*_0_ are the absorbance values of the sample, the background control of the sample, and the blank control, respectively.

## 5. Conclusions

In conclusion, it was found that *Piperis longi fructus* is rich in compounds and has strong antioxidant activity. This newly developed method is applicable and effective for the large-scale industrial purification of total alkaloids with lower cost, higher efficiency, and procedural simplicity.

## Figures and Tables

**Table 1:** Adsorption kinetics of total alkaloids.

Adsorption Model	Parameters	D101
Pseudo-first-order kinetic model	*Q*_e_	485
	*K*_1_	1.99
	*R* ^2^	0.770
Pseudo-second-order kinetic model	*K*_2_	0.00881
	*R* ^2^	0.999
Particle internal diffusion model	*K*_d1_	49.1
	*C*_1_	151
	*R*_1_^2^	0.979
	*K*_d2_	4.37
	*C*_2_	435
	*R*_2_^2^	0.819

**Table 2:** Thermodynamic equation results of total alkaloids.

Temperature (*K*)	Langmuir Equation			Freundlich Equation		
	Q_m_	*K_L_*	*R* ^2^	*K_F_*	1/n	*R* ^2^
313	625	0.329	0.980	49.2	0.688	0.957
318	641	0.320	0.979	48.4	0.650	0.894
323	671	0.310	0.990	46.2	0.650	0.738
328	671	0.308	0.993	55.9	0.553	0.777

**Table 3:** Thermodynamic parameter results of total alkaloids.

*Q_e_* (mg/g)	Δ*H*^o^ KJ·mol^−1^)	Δ*G*^o^ (KJ·mol^−1^)				Δ*S*^o^ (KJ·mol^−1^·K^−1^)			
		313	318	323	328	313	318	323	328
1.00	27.1	−6.01	−7.23	−8.07	−8.72	0.110	0.108	0.106	0.105
1.50	48.2					0.180	0.177	0.174	0.172
2.00	54.8					0.201	0.198	0.195	0.192
2.50	58.5					0.215	0.211	0.208	0.205

## Data Availability

The data presented in this study are available within the article.

## Supplementary Materials

The following supporting information can be downloaded at https://www.mdpi.com/article/10.3390/molecules30071476/s1. Supplementary material in the paper Figure S1 and Tables S1–S3. Supplementary Materials Figure S1: Freeze-dried powder; Supplementary Materials Table S1: Identification of 44 compounds in Piperis longi fructus; Supplementary Materials Table S2: Comparison of antioxidant activity in Piperis longi fructus; Supplementary Materials Table S3: Detailed information of different macroporous resin.
