# Supplementary material for: Purification and Oxidative Scavenging of Total Alkaloids of Piperis longi fructus Based on Adsorption Kinetics and Thermodynamic Theory
Source: Molecules. 2025 Mar 26;30(7):1476. doi: 10.3390/molecules30071476 (PMC11990382; doi:10.3390/molecules30071476)
Supplement: Supplementary file 1 [file molecules-30-01476-s001.zip › molecules-3450118 - supplementary/Supplementary Materials Table S1.pdf]

Table S1. Identification of 44 compounds in Piperis Longi Fructus

No.	t_R (min)	Compound	$[M+H]^+$	Error (ppm)	MS^2	MF
1	7.15	Threo-1-[1-oxo-9(3,4-methylenedioxyphenyl)-8,9-dihydroxy-2E-nonenyl]-piperidine	376.2112	-1.7	358.2004,135.0439	$C_{21}H_{29}NO_5$
2	7.15	Erythro-1-[1-oxo-9(3,4-methylenedioxyphenyl)-8,9-dihydroxy-2E-nonenyl]-piperidine	376.2112	-1.7	135.0439,358.2004	$C_{21}H_{29}NO_5$
3	7.54	Fagaramide	248.1279	-1	175.0392	$C_{14}H_{17}NO_3$
4	7.94	Piperyline	272.1279	-1	135.0440,201.0557	$C_{16}H_{17}NO_3$
5	8.54	4,5-Dihydropiperlonguminine	276.1592	-0.7	135.0489	$C_{16}H_{21}NO_3$
6	8.63	Piperlonguminine	274.1435	-1	135.0476,201.0577	$C_{16}H_{19}NO_3$
7	8.82	Piperanine	288.1593	-0.3	135.0485	$C_{17}H_{21}NO_3$
8	9.00	Piperine	286.1435	-0.9	201.0566	$C_{17}H_{19}NO_3$
9	10.12	Piperdardine	314.1750	-0.4	135.0439	$C_{19}H_{23}NO_3$
10	10.29	Piperettine I	312.1592	-0.6	169.0650,227.0699	$C_{19}H_{21}NO_3$
11	10.29	Piperettine Ⅱ	312.1592	-0.6	169.0650,227.0699	$C_{19}H_{21}NO_3$
12	10.29	Piperettine Ⅲ	312.1592	-0.6	169.0650,227.0699	$C_{19}H_{21}NO_3$
13	10.29	Piperettine IV	312.1592	-0.6	169.0650,227.0699	$C_{19}H_{21}NO_3$
14	10.77	Retrofractamide A	328.1906	-0.5	161.0600,255.1016,227.1060	$C_{20}H_{25}NO_3$
15	10.77	Brachyamide B	328.1906	-0.5	161.0600	$C_{20}H_{25}NO_3$
16	10.80	Pellitorine	224.2006	-1.1	224.2034,168.1399	$C_{14}H_{25}NO$
17	10.89	Pipgulzarine	372.2515	-5	161.0586	$C_{23}H_{33}NO_3$
18	11.09	Pipercallosine	330.2061	-0.9	135.0440,229.1229	$C_{20}H_{27}NO_3$
19	11.21	Piptigrine	340.1904	-0.9	227.1063	$C_{21}H_{25}NO_3$
20	11.21	Dehydropipernonaline	340.1904	-0.9	131.0489,117.2063	$C_{21}H_{25}NO_3$
21	11.31	Dehydroretrofractamide C	332.2217	-0.9	135.0437	$C_{20}H_{29}NO_3$
22	11.54	N-isobutyl-2E,4E-undecadienamide	238.2164	-0.5	168.1369	$C_{15}H_{27}NO$
23	11.58	Pipernonaline	342.2062	-0.4	135.0459,229.1245	$C_{21}H_{27}NO_3$

24	12.02	Piperolein B	344.2217	-0.9	135.0439	$C_{21}H_{29}NO_3$
25	12.12	Retrofractamide B	356.2219	-0.5	135.1438,255.1381	$C_{22}H_{29}NO_3$
26	12.49	Piperchabamide D	358.2371	-1.7	135.0438,285.1468	$C_{22}H_{31}NO_3$
27	12.68	Piperundecalidine	368.2217	-0.9	135.0440,255.1378	$C_{23}H_{29}NO_3$
28	13.11	Piperchabamide B	370.2368	-2.3	135.0435	$C_{23}H_{31}NO_3$
29	13.46	Guineensine	384.2530	-0.9	135.0438,283.1696	$C_{24}H_{33}NO_3$
30	13.99	(2E,4E,13E)-14-(Benzo[d][1,3]dioxol-6-yl)-N-isobutyltetrade-ca-2,4,13-trienamide	398.2692	0.6	135.0446	$C_{25}H_{35}NO_3$
31	14.08	(2E,4E,12E)-13-(Benzo[d][1,3]dioxol-6-yl)-1-(piperidin-1-yl)trideca-2,4,12-trien-1-one	396.2533	0	135.0431	$C_{25}H_{33}NO_3$
32	14.76	Brachystamide B	412.2830	-3.9	135.0437,339.2123	$C_{26}H_{37}NO_3$
33	14.87	1-(piperidinyl)-2,8-tetradecadien-1-one	292.2635	-0.1	292.2641	$C_{19}H_{33}NO$
34	14.87	N-[(2E,4E)-Tetradecadienoyl]piperidine	292.2635	-0.1	292.2641	$C_{19}H_{33}NO$
35	15.64	N-Isobutyl-2E,4E-hexadecadienamide	308.2943	-1.7	308.2948	$C_{20}H_{37}NO$
36	15.81	(2E,4E,12Z)-N-Isobutylocatadeca-2,4,12-trienamide	334.3099	-1.6	334.3136	$C_{22}H_{39}NO$
37	16.50	1-(2E,4E,12E)-octadecatrinoylpiperidine	346.3102	-0.9	346.3105	$C_{23}H_{39}NO$
38	16.94	N-Isobutyl-(2E,4E)-octadecadienamide	336.3255	-1.9	336.3253	$C_{22}H_{41}NO$
39	17.03	(2E,4E,15Z)-N-isobutyl-eicosa-2,4,15-trienamide	362.3412	-1.4	362.3416	$C_{24}H_{43}NO$
40	17.05	1-(piperidinyl)-2,4-octadecadien-1-one	348.3255	-1.6	348.3095	$C_{23}H_{41}NO$
41	17.51	N-isobutyl-2E,4E-decyldecadienamide	364.3553	-5.8	291.2744	$C_{24}H_{45}NO$

42	18.02	1-[(2E,4E,14Z)-1-oxo-2,4,14-eicosatrienyl]-piperidine	374.3400	-4.8	374.3412	$C_{25}H_{43}NO$

The structural formulas of the above 42 compound components.

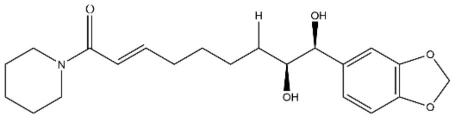

[1] Threo-1-[1-oxo-9(3,4-methylenedioxyphenyl)-8,9-dihydroxy-2E-nonenyl]-piperidine

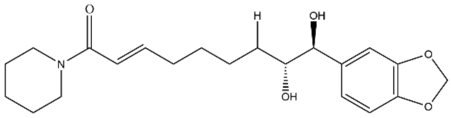

[2] Erythro-1-[1-oxo-9(3,4-methylenedioxyphenyl)-8,9-dihydroxy-2E-nonenyl]-piperidine

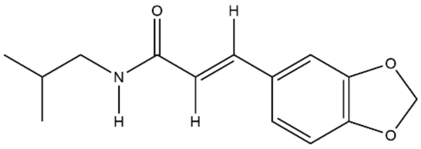

[3] Fagaramide

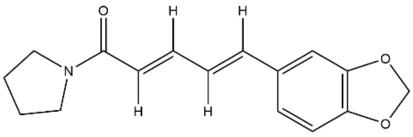

[4] Piperyline

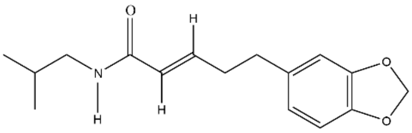

[5] 4,5-Dihydropiperlonguminine

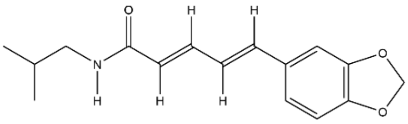

[6] Piperlonguminine

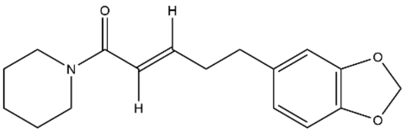

[7] Piperanine

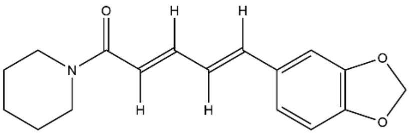

[8] Piperine

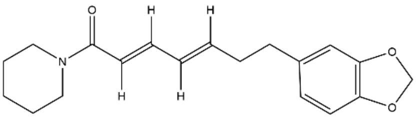

[9] Piperdardine

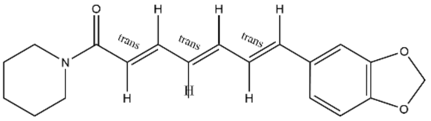

[10] Piperettine I

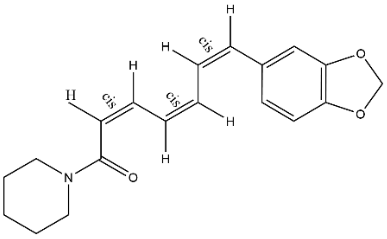

[11] Piperettine II

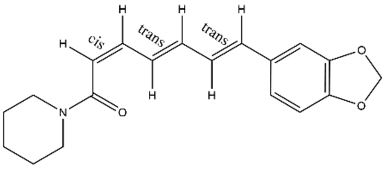

[12] Piperettine III

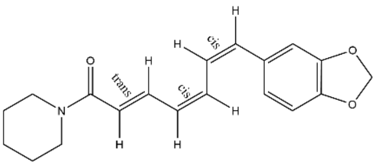

[13] Piperettine IV

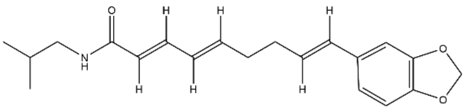

[14] Retrofractamide A

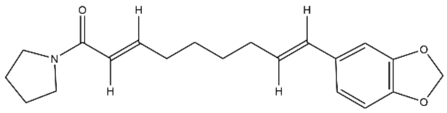

[15] Brachyamide B

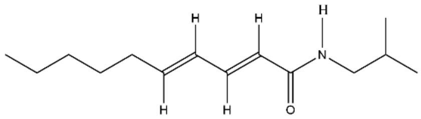

[16] Pellitorine

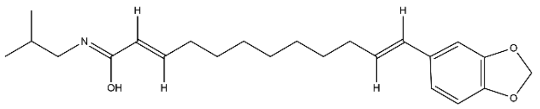

[17] Pipgulzarine

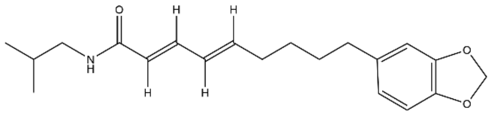

[18] Pipercallosine

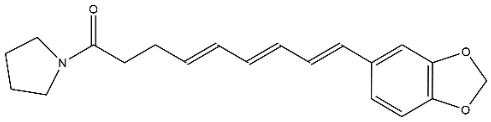

[19] Piptigrine

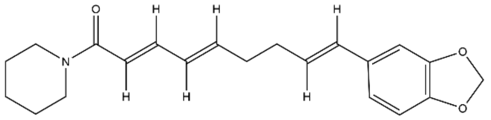

[20] Dehydropipernonaline

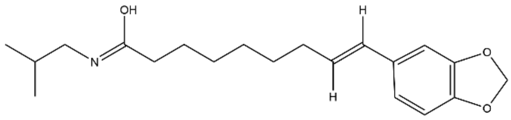

[21] Dehydroretrofractamide C

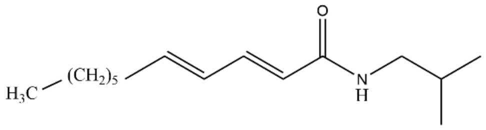

[22] N-isobutyl-2E,4E-undecadienamide

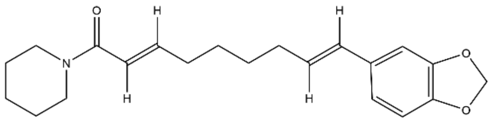

[23] Pipernonaline

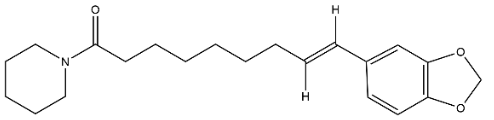

[24] Piperolein B

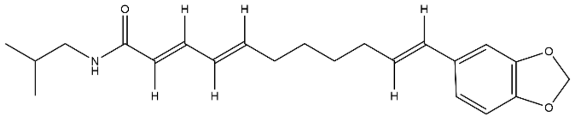

[25] Retrofractamide B

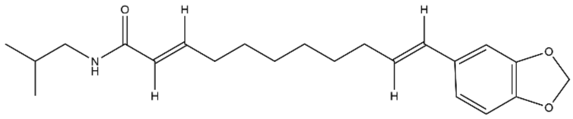

[26] Piperchabamide D

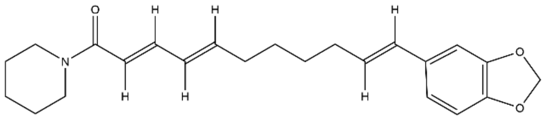

[27] Piperundecalidine

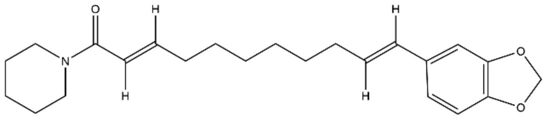

[28] Piperchabamide B

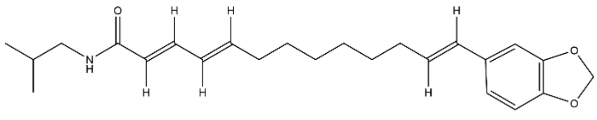

[29] Guineensine

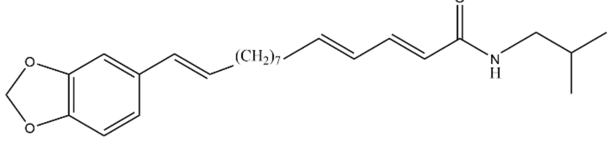

[30] (2E,4E,13E)-14-(Benzo[d][1,3]dioxol-6-yl)-N-isobutyltetrade-ca-2,4,13-trienamide

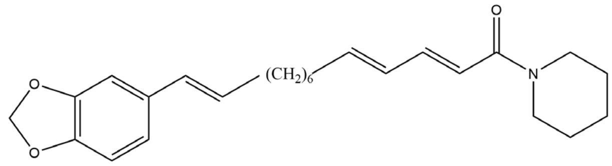

[31] (2E,4E,12E)-13-(Benzo[d][1,3]dioxol-6-yl)-1-(piperidin-1-yl)trideca-2,4,12-trien-1-one

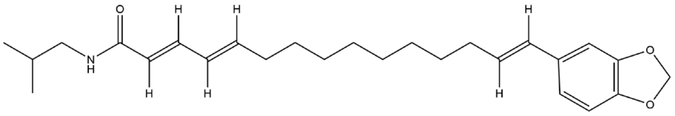

[32] Brachystamide B

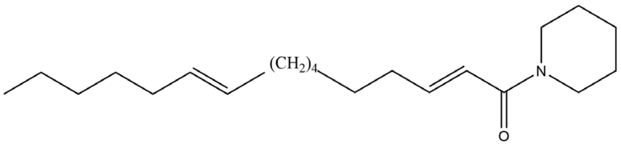

[33] 1-(piperidinyl)-2,8-tetradecadien-1-one

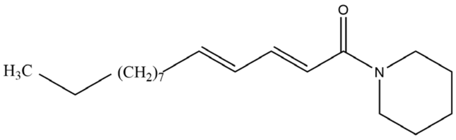

[34] N-[(2E,4E)-Tetradecadienoyl]piperidine

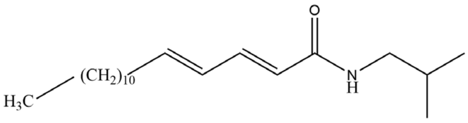

[35] N-Isobutyl-2E,4E-hexadecadienamide

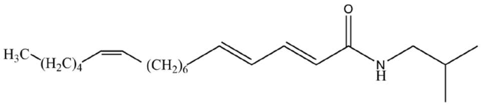

[36] (2E,4E,12Z)-N-Isobutylocatadeca-2,4,12-trienamide

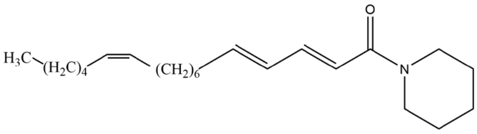

[37] 1-(2E,4E,12E)-octadecatrinoylpiperidine

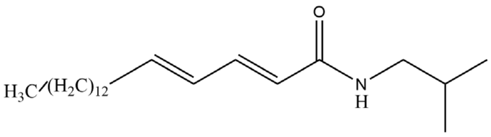

[38] N-Isobutyl-(2E,4E)-octadecadienamide

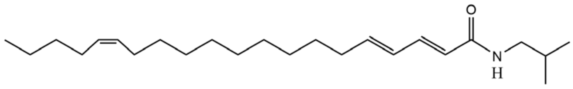

[39] (2E,4E,15Z)-N-isobutyl-eicosa-2,4,15-trienamide

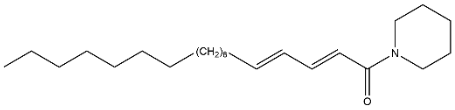

[40] 1-(piperidinyl)-2,4-octadecadien-1-one

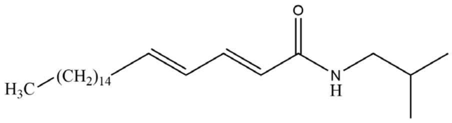

[41] N-isobutyl-2E,4E-decyldecadienamide

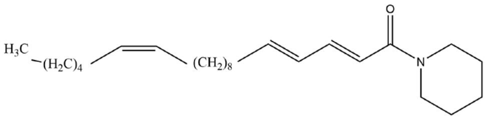

[42] 1-[(2E,4E,14Z)-1-oxo-2,4,14-eicosatrienyl]-piperidine
