# Supplementary material for: Purification and Oxidative Scavenging of Total Alkaloids of Piperis longi fructus Based on Adsorption Kinetics and Thermodynamic Theory
Source: Molecules. 2025 Mar 26;30(7):1476. doi: 10.3390/molecules30071476 (PMC11990382; doi:10.3390/molecules30071476)
Supplement: Supplementary file 1 [file molecules-30-01476-s001.zip › molecules-3450118 - supplementary/Supplementary Materials Table S2.pdf]

Table S2. Comparison of antioxidant activity in Piperis Longi Fructus

**Index**	**Regression equations**	
	Alcohol extracts	Purified substances
DPPH	$Y = 27.4566x + 40.1452$, $R^2 = 0.9375$	$Y = 26.8742x + 43.5049$, $R^2 = 0.9428$
$ABTS^+$	$Y = 25.4395x + 12.5911$, $R^2 = 0.9706$	$Y = 26.9358x + 15.4702$, $R^2 = 0.9741$
DPPH IC_{50} (mg/mL)	0.36	0.24
$ABTS^+$ IC_{50} (mg/mL)	1.47	1.28
