# Supplementary material for: Purification and Oxidative Scavenging of Total Alkaloids of Piperis longi fructus Based on Adsorption Kinetics and Thermodynamic Theory
Source: Molecules. 2025 Mar 26;30(7):1476. doi: 10.3390/molecules30071476 (PMC11990382; doi:10.3390/molecules30071476)
Supplement: Supplementary file 1 [file molecules-30-01476-s001.zip › molecules-3450118 - supplementary/Supplementary Materials Table S3.pdf]

Table S3. Detailed information of different macroporous resin.

Name	Polarity	Surface area (m^2/g)	Average pore diameter (Å)	Particle diameter (mm)	Water content (%)
D101	Non-polar	550-600	90-100	0.3-1.25	65-75
AB-8	Low-polar	480~520	100-120	0.3-1.25	65-75
HP-20	Non-polar	500-600	290-300	0.3-1.25	65-75
S-8	Polar	550-600	90-100	0.3-1.25	65-75
HPD-300	Medium-polar	570	240	0.355	55-65
NKA-9	Polar	500-550	100-120	0.3-1.25	65-75
X-5	Non-polar	500~600	290-300	0.3-1.25	79-80
