# Supplementary figures and images for: Purification and Oxidative Scavenging of Total Alkaloids of Piperis longi fructus Based on Adsorption Kinetics and Thermodynamic Theory
Source: Molecules. 2025 Mar 26;30(7):1476. doi: 10.3390/molecules30071476 (PMC11990382; doi:10.3390/molecules30071476)

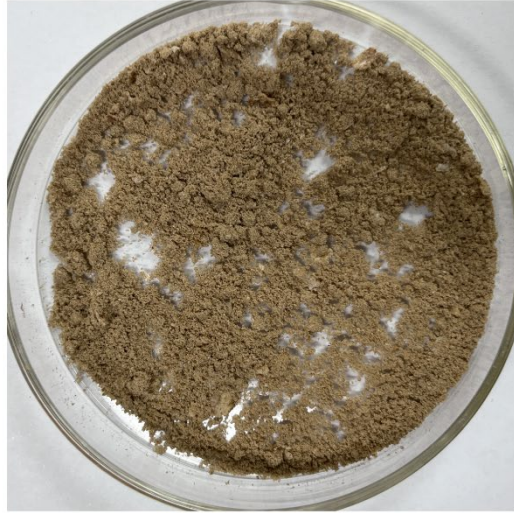

**Figure S1.** Freeze-dried powder

Supplement: Supplementary file 1 [file molecules-30-01476-s001.zip › molecules-3450118 - supplementary/Supplementary Materials Figure S1.pdf]
